# Adherence to the CLOSE Protocol and Low Baseline Generator Impedance Are Independent Predictors of Durable Pulmonary Vein Isolation

**DOI:** 10.3390/jcm13071960

**Published:** 2024-03-28

**Authors:** Márton Boga, Gábor Orbán, Péter Perge, Zoltán Salló, Edit Tanai, Arnold Béla Ferencz, Patrik Tóth, Ferenc Komlósi, István Osztheimer, Klaudia Vivien Nagy, Béla Merkely, László Gellér, Nándor Szegedi

**Affiliations:** Heart and Vascular Center, Semmelweis University, 1085 Budapest, Hungary; bogamarci99@gmail.com (M.B.); orbn.gbor@gmail.com (G.O.);

**Keywords:** pulmonary vein isolation, pulmonary vein reconnection, CLOSE protocol, generator impedance

## Abstract

**Background:** Atrial fibrillation (AF) recurrence after pulmonary vein isolation (PVI) is predominantly attributed to pulmonary vein reconnection (PVR). Predictors of AF recurrence have been widely studied; however, data are scarce on procedural parameters that predict chronic PVR. We aimed to study PVR rates and predictors of PVR. **Methods:** We retrospectively included 100 patients who underwent repeated ablation due to AF recurrence after initial PVI with the CARTO system. PVR was determined during the repeated procedure by electrophysiological evaluation, and initial procedural characteristics predicting PVR were studied, including adherence to the CLOSE protocol, use of high power, first-pass isolation (FPI), and baseline generator impedance (BGI). **Results:** Thirty-eight patients underwent initial CLOSE-guided PVI, and sixty-two underwent initial non-CLOSE PVI. A repeat procedure was performed 23 ± 16 months after the initial procedure. In total, PVR was found in 192 of 373 PVs (51.5%), and all PVs were isolated in 17/100 (17%) patients. Factors associated with all PVs being isolated were adherence to the CLOSE protocol, a higher power setting, the presence of bilateral FPI, and lower BGI (88% vs. 28%, *p* < 0.0001; 37.5 W vs. 30 W, *p* = 0.0276; 88.2% vs. 40.4%, *p* = 0.0007; and 127.6 Ω vs. 136.6 Ω, *p* = 0.0027, respectively). In initial procedures with adherence to the CLOSE protocol, the FPI rate was significantly higher (73.7% vs. 25%, *p* < 0.0001), while there were no significant differences in terms of procedure time and left atrial dwell time (81 vs. 85 min, *p* = 0.83; and 60 vs. 58 min, *p* = 0.08, respectively). BGI ≥ 130 Ω (AUC = 0.7403, sensitivity: 77.1%, specificity: 68.8%, *p* = 0.0032) was associated with a significantly higher probability of PVR (OR = 6.757; *p* < 0.0001). In multivariable analysis, independent predictors for PVR were non-adherence to the CLOSE protocol and BGI ≥ 130 Ω. **Conclusions:** Our findings indicate that adherence to the CLOSE protocol and baseline generator impedance < 130 Ω during AF ablation are independent predictors of PVI durability.

## 1. Introduction

Atrial fibrillation (AF) is the most common sustained cardiac arrhythmia, associated with an increase in morbidity, mortality, and deterioration in the quality of life [1]. Pulmonary vein isolation (PVI) is the cornerstone of AF management, and one of the most widely used techniques for PVI is point-by-point radiofrequency (RF) ablation [2]. Although acute isolation of all pulmonary veins (PVs) is achieved in nearly all cases, recurrent atrial tachyarrhythmias (ATAs) following AF ablation are frequent [2,3]. These recurrences are predominantly attributed to pulmonary vein reconnection (PVR), suggesting either inadequate lesion formation or discontinuity in the ablation line during the initial procedure [4].

The predictors of AF recurrence are well known [5,6,7,8]; however, procedural parameters predicting chronic PVR are not clearly established. The CLOSE protocol is an RF ablation strategy designed to encircle the PVs with continuous and optimized radiofrequency lesions. This approach involves a target inter-lesion distance (ILD) of ≤6 mm, along with an ablation index (AI) of ≥400 at the posterior wall and ≥550 at the anterior wall [9]. CLOSE protocol-guided PVI results in outstanding rates of first-pass isolation (FPI) and 12-month freedom from ATA, as well as a significant decrease in ATA burden [10,11]. However, there is a lack of data comparing the durability of PVI with and without adherence to the CLOSE approach, as assessed by a repeated electrophysiological evaluation [12]. Previous findings indicate that successful first-pass isolation after creating a circumferential ablation line around the PVs is associated with improved ablation outcomes [3]. It has also been implied that baseline generator impedance (BGI) of ablation points influences the effectiveness of RF ablation [13,14,15]; however, its effect on PVI durability has never been assessed before. Therefore, we aimed to assess the incidence of PVR during repeated procedures and to conduct an in-depth analysis of the predictors of PVR, including non-adherence to our institutional CLOSE protocol (ILD ≤ 5 mm, posterior AI ≥ 400, anterior AI ≥ 500), absence of FPI, and BGI of ablation points. Hereinafter, reference to the CLOSE protocol indicates the CLOSE criteria used in our electrophysiology laboratory as defined above.

## 2. Materials and Methods

**Figure 1:**
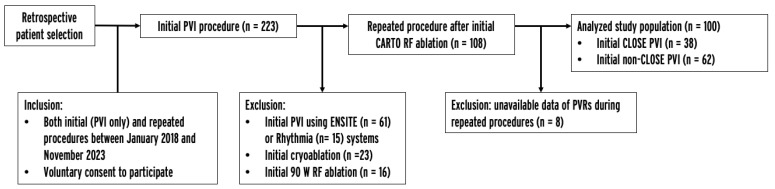
Study enrollment flowchart. PVI = pulmonary vein isolation, PVR = pulmonary vein reconnection, RF = radiofrequency.

The current single-center, retrospective, observational study included patients who underwent their first PVI procedure with the CARTO system between January 2018 and November 2023 and a repeated procedure in the same period. Data were obtained from an institutional AF ablation registry. Patients were excluded if the initial procedure was performed using the ENSITE or Rhythmia system, 90 W RF ablation, or cryoablation, as the CLOSE protocol, by definition, was not possible in these cases. Also, the protocol mandated repeated procedures (as part of a remap study), and the unavailability of data about PVRs during repeated procedures led to exclusion from the study. The inclusion and exclusion criteria are listed in the study enrollment flowchart (Figure 1). All patients provided written informed consent to the ablation procedure, data retrieval, and analysis. Ethics approval was obtained from the Semmelweis University Regional and Institutional Committee of Science and Research Ethics.

### 2.1. Procedural Parameters and Study Endpoints

We recorded the following procedural parameters: BGI of ablation points, first-pass isolation, mean PV-pair perimeter (measured in the CARTO system), power setting, number of RF applications, procedure time, left atrial dwell time, fluoroscopy time, dose area product, time to AF recurrence, and time to repeated procedure after initial PVI. The primary endpoint of the current study was pulmonary vein reconnection at the repeat procedure.

### 2.2. Initital PVI Procedure

**Figure 2:**
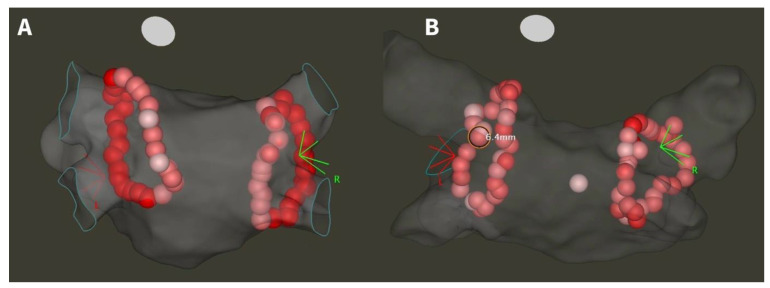
Anatomical map of the left atrium after CLOSE-guided (**A**) and non-CLOSE PVI (**B**). White tags indicate AI < 400, pink tags AI 400–500, and red tags AI > 500. ILD < 5 mm at each site in the case of CLOSE adherence, and >5 mm at some sites in the case of non-CLOSE PVI.

Before the procedure, all patients underwent either contrast-enhanced left atrial computed tomography or transesophageal echocardiography in order to exclude left atrial appendage thrombus. The catheter ablation was conducted under conscious sedation. Procedures were performed by six experienced operators (each performing >100 PVI/year). Following femoral venous access, double transseptal puncture was guided by fluoroscopy and pressure monitoring. Intravenous heparin was administered according to body weight, with dosage adjustments aimed at reaching an activated clotting time of ≥300 s. Utilizing an electroanatomical mapping system (CARTO 3, Biosense Webster Inc., Diamond Bar, CA, USA) and a decapolar mapping catheter (Lasso, Biosense Webster Inc., Diamond Bar, CA, USA), an anatomical left atrial map was generated. Point-by-point PVI was performed using a steerable sheath and contact-force (CF) sensing ablation catheter (either SmartTouch or QDOT Micro, Biosense Webster Inc., Diamond Bar, CA, USA). Adherence to the CLOSE approach and power settings were based on the operating physician’s preference. The ablation power was set at either 30/35 W (low-power long-duration, LPLD) or 40/45/50 W (high-power short-duration, HPSD), and was not changed during a given ablation procedure. All operators performed both CLOSE and non-CLOSE PVI. The goal during CLOSE-guided procedures was to create wide circumferential lesion sets around the ipsilateral PVs targeting ILDs ≤ 5 mm, and AI ≥ 400 posteriorly and ≥500 anteriorly, while there was no strict protocol for the other group of patients, only to reach complete PVI at the end of the procedure. Adherence to the protocol was confirmed or ruled out based on examination of CARTO maps during the study period. Adherence to the CLOSE protocol was confirmed in cases where there was a continuous chain of applications with no ILD greater than 5 mm and for each application an AI of ≥400 posteriorly and ≥500 anteriorly was reached. Following the completion of the initial circle, the presence or absence of first-pass isolation was assessed using the multipolar catheter. In the case of incomplete isolation, further touch-up applications were delivered until complete PVI was achieved. A representative anatomical map of the left atrium after CLOSE and non-CLOSE PVI is shown in Figure 2. No additional arrhythmia substrates were targeted beyond PVI.

### 2.3. Repeat Procedure

Following the initial procedure, AF recurrence was monitored in all patients by standard of care follow-up, with 12-lead electrocardiograms and 24 h Holter monitoring at 3, 6, and 12 months, and in case of arrhythmia symptoms. All patients enrolled in this study had symptomatic AF recurrence, and underwent a repeated procedure. The procedure was performed under conscious sedation, using a decapolar Lasso or PentaRay (Biosense Webster Inc., Diamond Bar, CA, USA) catheter and a CF-sensing ablation catheter. Pulmonary vein reconnection (PVR) was defined as the presence of near-field signals within the PVs detected by the multipolar mapping catheter. Upon identification of PVR, the reconnected vein(s) were re-isolated. In cases where all PVs were isolated at the beginning of the repeat procedure, patients underwent ablation, targeting non-PV triggers, and adjunctive substrate modification in persistent AF cases.

### 2.4. Statistics

Continuous variables are reported as either the mean and standard deviation or median and inter-quartile range. The distribution of variables was tested with the Shapiro–Wilk test. For the comparison of unpaired groups, the Student *t*-test was used for normally distributed parameters, and the Mann–Whitney U test was used for non-parametric data. Categorical variables are reported as frequency and percentage and were compared by Fisher’s exact test. Optimal cut-off values were established using receiver operating characteristic analysis. Log-rank analysis was used to compare survival times. To accurately assess the impact of different variables on PVI durability, we performed univariate and multivariable analyses. In univariate analysis, simple logistic regression was fitted to all variables for predicting ≥1 PVR. The variables that resulted in *p* < 0.1 in univariate analysis were included in the multivariable model. Statistical analyses were conducted using GraphPad Prism 10 (GraphPad Softwares Inc., San Diego, CA, USA). A significance level of *p* < 0.05 (two-tailed) was considered statistically significant.

## 3. Results

### 3.1. Study Population

Overall, 100 patients met the inclusion and exclusion criteria. Table 1 presents an overview of the baseline characteristics of the study population. Patients had a mean age of 60 ± 12 years, 36% were female, and 44% had persistent AF. Thirty-eight patients underwent initial PVI with strict adherence to the CLOSE protocol, and sixty-two patients underwent initial non-CLOSE PVI. There was no significant difference in terms of baseline characteristics between patients treated with CLOSE and non-CLOSE PVI.

### 3.2. Incidence of PVR

Eighty-two initial procedures were performed using the SmartTouch catheter, and 16 procedures were performed with the QDOT Micro catheter with the 50 W energy setting. Thirty procedures were performed using a high power setting (HPSD, 40–50 W) and 70 procedures were performed with a low power setting (LPLD, 30–35 W).

The repeated procedure was performed 23 ± 16 months after the initial procedure. The isolation of 200 PV pairs and 373 PVs at the repeat procedure were studied. In total, PVR was found in 192 of 373 PVs (51.5%). The locations of the PVRs were as follows: left superior PV—42 (21.9%), left inferior PV—37 (19.3%), left common trunk—7 (3.6%), right superior PV—56 (29.2%), right inferior PV—53 (27.6%), right middle PV—5 (2.6%). All PVs were isolated in 17/100 patients at the repeated procedure.

**Figure 3:**
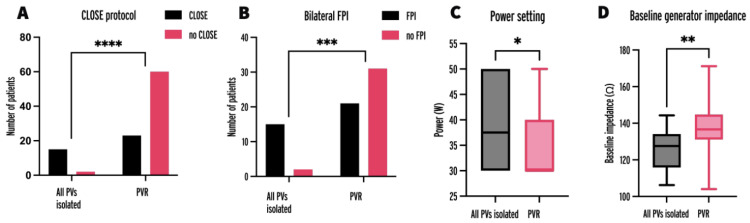
Number of initial procedures with adherence to the CLOSE protocol (**A**), bilateral FPI (**B**), power setting (**C**), and baseline generator impedance (**D**) in procedures leading to all PVs being isolated and at least one PVR at repeat procedure. FPI = first-pass isolation; PV = pulmonary vein; PVR = pulmonary vein reconnection; * = *p* < 0.05, ** = *p* < 0.01, *** = *p* < 0.001, **** = *p* < 0.0001.

The parameters of patients and initial procedures with all PVs isolated were compared to patients and procedures with at least one PVR (Table 2). Factors significantly associated with all PVs being isolated were adherence to the CLOSE protocol (Figure 3A), 88% vs. 28%, *p* < 0.0001; presence of FPI (Figure 3B) 88.2% vs. 40.4%, *p* = 0.0007; higher power setting (Figure 3C), 37.5 W vs. 30 W, *p* = 0.0276; and lower BGI (Figure 3D), 127.6 Ω vs. 136.6 Ω, *p* = 0.0027.

The time to AF recurrence and the time to repeated procedure after initial PVI did not differ significantly between patients, with all PVs isolated and patients with PVR (17.8 vs. 15.5 months, HR = 0.7426, *p* = 0.2630; and 23.8 vs. 18.3 months, HR = 0.7081, *p* = 0.1814).

### 3.3. CLOSE vs. Non-CLOSE PVI

Initial PVI with adherence to the CLOSE protocol resulted in significantly fewer PVRs on a per PV basis (26.1% vs. 68.3%, OR = 0.16, 95% CI = 0.10–0.26, *p* < 0.0001), and the number of patients with all PVs isolated was significantly higher compared to non-CLOSE PVI (39.5% vs. 3.5%, OR = 18.26, 95% CI = 2.00–4.47, *p* < 0.0001). Comparing the procedural characteristics of the initial procedure with and without adherence to the CLOSE protocol (Table 3), there was a significant difference in fluoroscopy time, dose area product, number of RF applications, and FPI rate (4.7 vs. 5.8 min, *p* =0.0467; 152.6 vs. 232 uGym^2^, *p* = 0.0334; 72 vs. 82, *p* = 0.0202; 73.7% vs. 25%, *p* < 0.0001, respectively), while there were no significant differences in terms of the procedure time, left atrial dwell time, and baseline generator impedance (81 vs. 85 min, *p* = 0.83; 60 vs. 58 min, *p* = 0.08; 131.7 vs. 136.3 Ω, *p* = 0.25, respectively).

### 3.4. Baseline Generator Impedance

**Figure 4:**
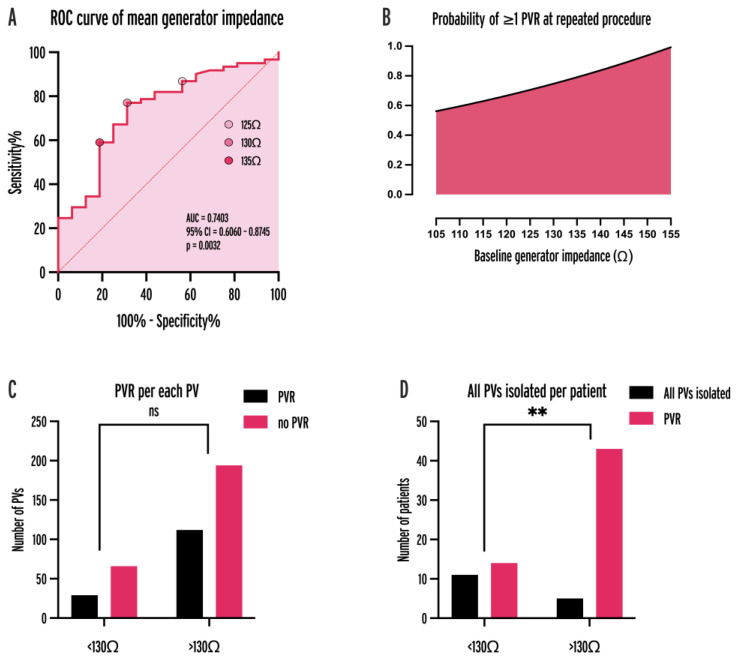
ROC curve of mean baseline generator impedance for predicting PVR (**A**), probability of ≥1 PVR as a fitted non-linear function of baseline impedance (**B**), PVR per each PV (**C**), and all PVs isolated per patient (**D**) for 130 Ω cut-off. AUC = area under curve, CI = confidence interval, PV = pulmonary vein, PVR = pulmonary vein reconnection, ROC = receiver operator characteristic, **: *p* < 0.001.

We analyzed the BGI of 6562 ablation points, the mean of which was calculated for each procedure. In procedures with at least one PVR, the mean BGI was significantly higher (127.6 vs. 136.6 Ω, *p* = 0.0027). To identify the optimal BGI threshold for predicting PVR, a receiver operating characteristic (ROC) analysis was performed (AUC = 0.7403, 95% CI = 0.6060–0.8745, *p* = 0.0032; Figure 4A). Baseline impedance of 130 Ω was an optimal cut-off (sensitivity: 77.1%, specificity: 68.8%, positive predictive value: 68.75%, negative predictive value: 75.44%). The probability of at least one PVR was higher in the case of initial procedures with mean baseline impedance ≥ 130 Ω (OR = 6.757, *p* < 0.0001; Figure 4D).

### 3.5. First-Pass Isolation

Data on the presence of bilateral FPI were recorded during 70 initial PVI procedures. Bilateral FPI resulted in fewer PVRs on a per PV basis, and a greater probability of all PVs being isolated per patient (OR = 0.32, *p* < 0.0001; and OR = 7.26, *p* = 0.0056). Bilateral FPI was associated with adherence to the CLOSE protocol (77.8% vs. 29.4%, *p* < 0.0001), a higher power setting (35 vs. 30 W, *p* = 0.0324), a lower mean PV-pair perimeter (11.9 vs. 12.8 cm, *p* = 0.0233), fewer RF applications (72 vs. 87, *p* = 0.0077), and lower BGI (131.2 vs. 136.7 Ω, *p* = 0.0487). A per side analysis also showed that FPI of left PVs and FPI of right PVs led to a smaller probability of PVR per PV (OR = 0.15 for left PVs; and OR = 0.14 for right PVs) and a greater probability of all PVs being isolated on the same side (OR = 19.18 for left PVs; and OR = 15.74 for right PVs).

### 3.6. Multivariable Analysis for Predictors of PVR

Variables that resulted in *p* < 0.1 in univariate analysis (Table 4) for predicting ≥1 PVR and PVR/PVs per patient were the same for the two endpoints: adherence to the CLOSE protocol, a high power setting (40–50 W HPSD), the presence of bilateral FPI, BGI ≥ 130 Ω, and catheter type (QDOT). The variable power setting was dichotomized to 30–35 W LPLD and 40–50 W HPSD categories and mean BGI per patient was dichotomized to ≥130 Ω and <130 Ω categories. Two initial procedures were excluded from the analysis because of missing data. A multivariable logistic regression model was fitted including the above variables for predicting ≥1 PVR per patient (Table 5; AUC = 0.9004, 95% CI = 0.8159–0.9850, positive predictive power = 83.33%, negative predictive power = 87.23%). Independent predictors for at least one PVR were adherence to the CLOSE protocol (OR = 0.0546, 95% CI = 0.0024–0.4456, *p* = 0.0187) and BGI ≥ 130 Ω (OR = 16.09, 95% CI = 2.089–220.3, *p* = 0.0157).

## 4. Discussion

### 4.1. Main Message

The results of this study show that strict adherence to the CLOSE protocol, utilization of higher power settings, achieving FPI, and lower baseline generator impedance during AF ablation enhance the durability of PVI and reduce the incidence of PVR. Importantly, the efficacy benefit in CLOSE procedures is not accompanied by prolonged procedure times. Furthermore, BGI ≥ 130 Ω is a new independent predictor of PVI durability in multivariable analysis, thereby presenting a potential way to ultimately improve the long-term efficacy of AF ablation.

### 4.2. CLOSE Protocol and Repeat Procedure Studies

In the last decade, the introduction of CF sensing has allowed the monitoring of catheter tip–tissue contact to create more effective RF lesions. The EFFICAS I and II studies underscored the importance of maintaining adequate CF during ablation [16,17]. CF is an integral component of lesion indices, such as the Ablation Index (AI) utilized by the CARTO system, which incorporates CF, power, and ablation time in a weighted formula. AI is a crucial element of the CLOSE criteria, defined based on a study by El Haddad et al., analyzing parameters of the weakest links in the ablation chains of PVI procedures [4]. While the original target values may not universally predict optimal lesion formation, subsequent studies overwhelmingly support the protocol’s effectiveness regarding arrhythmia recurrence, reporting impressive first-pass isolation rates (82–98%) and 12-month freedom from ATA (78–91%) [9,11]. Two comparative studies have shown superior clinical efficacy of PVI with vs. without the CLOSE approach (94% vs. 84% and 79% vs. 64%) [12,18]. Based on this evidence, the CLOSE approach currently seems to be the optimal RF ablation strategy for the treatment of AF. Although AF-recurrence-free survival represents the clinical success of the treatment, the correct assessment of this endpoint is highly dependent on the follow-up methods. Furthermore, freedom from AF does not directly translate to PVI durability. In contrast, electrophysiological assessment of PVR during repeat procedures is the most accurate endpoint in terms of PVI’s long-term durability. To the best of our knowledge, no previous studies have compared the long-term durability of PVI with and without adherence to the CLOSE protocol by electrophysiological evaluation on a well-powered sample size.

In the study by Phlips et al., 10 patients underwent repeated ablation after ATA recurrence [12]. Out of these, three patients received CLOSE-guided and seven patients received non-CLOSE initial PVI. They reported 23 sites of reconnection in 18 PVs in the non-CLOSE group and 7 sites of reconnection in 7 PVs in the CLOSE patients. This sample size was too low to draw any conclusions on the long-term durability of CLOSE PVI. De Pooter et al. reported the results of 45 repeated ablations after initial CLOSE-guided ablation [19]. They found four isolated veins in 62% of patients, characterized by a higher incidence of low voltage in the LA; indicating that non-PVR-mediated AF recurrence may be more common in patients treated according to the CLOSE protocol. In another study, Pedrote et al. enrolled 21 patients, in whom 63 out of 336 PV segments showed reconnection, characterized by lower AI values [20].

In our study, we compared the results of 38 patients who received initial CLOSE ablation with 62 patients who received initial non-CLOSE PVI. Non-adherence to the CLOSE protocol resulted in about an 18 times higher probability of at least one PV being reconnected during repeated procedures, while adjusting for all other variables. Compared to the study by Phlips et al., the ability to show this outcome can be attributed to the greater sample size. De Pooter et al. found a higher incidence of all PVs being isolated at repeat procedures compared to our study in the case of CLOSE PVI (62% vs. 40%) [19]. The following reasons could explain this difference: (i) a longer time to repeat procedure (mean 23.5 months in our population vs. 11 months); (ii) a high percentage of persistent AF (60% in our population vs. 0%); (iii) a higher prevalence of comorbidities in our cohort [19]. In the present study, the total procedure duration did not differ significantly between initial procedures with and without the CLOSE approach. In non-CLOSE procedures, FPI was less commonly achieved and the number of RF applications was higher, possibly due to the need for further ablation at the gaps left in the first-pass circle. These results show that there is no downside to CLOSE-PVI in terms of being time-consuming. The fluoroscopy times and dose–area product were substantially lower in the CLOSE procedures, which may be attributed to the main reliance on the electroanatomic mapping system instead of fluoroscopy. These data on the effect of adhering to the CLOSE protocol strongly suggest that the protocol should be utilized during every RF PVI procedure; and thus, could provide evidence to further support the adoption of the CLOSE approach as standard practice.

### 4.3. First-Pass Isolation

Multiple investigations showed that the lack of FPI is associated with worse outcomes in terms of freedom from AF and increased incidence of PVR during repeated procedures [3,21,22,23]. Our univariate analysis results verify the impact of FPI on PVI durability on a greater sample size than previous studies. However, multivariable analysis did not identify FPI as an independent predictor of PVR. This may be attributed to the fact that reaching FPI depends on other variables, including adherence to the CLOSE protocol and BGI, which emerge as more robust predictors of PVI durability.

### 4.4. Higher Power Settings

Since the development of the CLOSE protocol, high-power short-duration (HPSD) ablation using a power setting of 40–50 W has been introduced. Although the CLOSE protocol was originally developed for low-power long-duration (LPLD) ablation (25–35 W), it has been validated for higher power settings as well. The POWER-AF randomized clinical trial aimed to compare CLOSE-guided HPSD ablation to LPLD ablation, reporting favorable procedure times for HPSD (82 vs. 100 min), with similar FPI rates and mid-term efficacy (89% vs. 87%, and 92% vs. 90%, respectively) [24]. Prospective, non-randomized studies have also shown the same results, along with comparable safety between the two groups [25]. In a recent prospective investigation by our research group, HPSD ablation with our institutional CLOSE protocol resulted in a higher FPI rate (78 vs. 57%) and lower 9-month AF recurrence rate (10 vs. 36%) compared to 30 W LPLD ablation [3]. It can be concluded from these studies that the CLOSE protocol criteria can be effectively and safely applied not only for low-power ablation, but also for HPSD ablation. In the present investigation, univariate analysis found that the use of higher power settings was associated with better PVI durability. However, multivariable analysis with the inclusion of power setting as a dichotomized variable (HPSD or LPLD) did not show the use of high power as an independent predictor of PVR. This could potentially be explained by the correction for catheter type (the QDOT catheter was only used in the 50 W HPSD setting).

### 4.5. Baseline Generator Impedance

The baseline generator impedance (BGI) is an RF ablation parameter representing the impedance of the RF circuit at the start of each application. The AI used by the CARTO system does not account for this parameter. Therefore, BGI is not integrated into the CLOSE protocol, leading to the oversight of this potentially important factor in defining the optimal approach to PVI. Multiple investigations have suggested that BGI could indeed affect the efficacy and safety of RF ablation. Lower generator impedance at identical power settings was shown to result in higher current delivery, resulting in larger lesion volumes and higher tissue temperatures in ex vivo models [13,14]. It was later confirmed by Bourier et al. in clinical settings that generator impedance plays a pivotal role in the amount of current delivered during an RF application and it varies significantly among patients, with lower levels observed in males and patients with a lower BMI [15]. Generator impedance is also dependent on and can be changed by the positioning of the neutral electrode which completes the RF circuit. In our recent study, we showed that the risk of silent cerebral embolism associated with RF ablation is substantially higher in case of BGI < 110 Ω during applications [26]. We proposed strategical positioning of the neutral electrode to achieve a target baseline impedance of ≥110 Ω before the first RF application in patients with lower initial generator impedance, aiming to mitigate the risk of cerebrovascular complications. Notably, the impact of baseline impedance on the efficacy of RF ablation has not been previously explored.

Our findings establish BGI as an independent predictor of the long-term durability of PVI, as BGI ≥ 130 Ω resulted in about a 16 times higher probability of at least one PV being reconnected, while adjusting for all other variables. Our hypothesis for explaining this observation is that out of two RF applications with similar AI, the one with a lower BGI (thus higher mean delivered current), may result in a larger and clinically more effective lesion. Therefore, it is advisable to position the neutral electrode to maintain a generator impedance value of 110–130 Ω to optimize both the efficacy and safety of RF PVI procedures.

### 4.6. Limitations

This was a single-center, retrospective investigation with six operating physicians; therefore, the sample size is moderate. However, to our knowledge, this is still the largest repeat procedure study on the subject. The patients treated with and without the CLOSE approach and with different power settings were based on the operating physician’s preference; however, there were no significant differences in the studied patients’ characteristics. We did not have a standardized study protocol mandating the use of only one catheter or power setting; nevertheless, adherence to the CLOSE protocol remained consistent across all types of ablation catheters and power settings. In order to account for the possible inhomogeneity of the initial procedures, we performed multivariable analysis to control for potential confounding variables selected in the model.

## 5. Conclusions

Our findings indicate that adherence to the CLOSE protocol and baseline generator impedance ≥ 130 Ω during AF ablation are independent predictors for the durability of PVI.

## Figures and Tables

**Table 1:** Baseline characteristics of the study population. There were no significant differences between CLOSE and non-CLOSE patients. AF = atrial fibrillation; BMI = body mass index; CAD = coronary artery disease; LAD = left atrial diameter; LVEF = left ventricular ejection fraction; PVI = pulmonary vein isolation; rePVI = repeated pulmonary vein isolation; TIA = transient ischemic attack. Continuous variables are reported as mean ± standard deviation, while categorical variables are reported as frequency and percentage.

		All Patients (*n* = 100)	CLOSE (*n* = 38)	No CLOSE (*n* = 62)	*p*-Value
Age, years		60 ± 12	60 ± 12	61 ± 12	0.8334
Sex, female (%)		36 (36)	16 (42)	20 (32)	0.3919
BMI, kg/m^2^		28.9 ± 5	28.5 ± 5	29.1 ± 5.2	0.5847
Persistent AF, *n* (%)		44 (44)	17 (45)	27 (44)	>0.9999
Hypertension, *n* (%)		70 (70)	28 (74)	42 (68)	0.6541
Diabetes, *n* (%)		18 (18)	6 (16)	12 (19)	0.7908
CAD, *n* (%)		16 (16)	5 (13)	11 (18)	0.5892
Prior stroke/TIA, *n* (%)		8 (8)	2 (5)	6 (10)	0.7066
LVEF, %	initial PVI rePVI	55.9 ± 9.3 53.8 ± 7.9	57.9 ± 5.8 54.5 ± 5.4	54.6 ± 11 53.4 ± 9.1	0.1281 0.6833
LAD, mm	initial PVI rePVI	49.1 ± 6.5 50.5 ± 6.7	49.4 ± 7.6 50.7 ± 7	48.9 ± 5.9 50.3 ± 6.6	0.7935 0.8339

**Table 2:** Initial procedural and patient characteristics in the case of all PVs being isolated and at least 1 PVR at the repeated procedure. Categorical variables are reported as frequency and percentage. Continuous variables are reported as median and inter-quartile range. Bold values indicate significance at *p* < 0.05. AF = atrial fibrillation, BMI = body mass index, FPI = first-pass isolation, OR = odds ratio, PV = pulmonary vein, PVR = pulmonary vein reconnection, ST = SmartTouch.

		All Veins Isolated (*n* = 17)	At Least 1 PVR (*n* = 83)	Comparative Analysis	
				*p*-Value	OR
CLOSE protocol, *n* (%)		15 (88)	23 (28)	**<0.0001**	0.0511
Catheter, *n* (%)	ST QDOT	12 (71) 5 (29)	70 (86) 11 (14)	0.1454	0.3771
Power setting, W		37.5 (30–50)	30 (30–40)	**0.0276**	
FPI, *n* (%)		15 (88.2)	21 (40.4)	**0.0007**	0.0903
Mean PV-pair perimeter, cm		12.18 (11.38–12.9)	12.7 (11.35–13.55)	0.2582	
Baseline generator impedance, Ω		127.6 (115.8–134.1)	136.6 (131.1–144.8)	**0.0027**	
Time to first recurrence after initial PVI, months		17.8 (6.2–37.3)	15.5 (5.6–27.8)	0.3042	
Time to repeated procedure, months		23.8 (6.9–44.5)	18.3 (11.2–32.5)	0.4378	
Age, years		66 (54–75)	62 (52–70)	0.4001	
Sex, female (%)		6 (35)	47 (56)	0.1198	2.394
BMI, kg/m^2^		27.8	29.1	0.3330	
Persistent AF, *n* (%)		8 (47)	36 (44)	0.7948	1.160

**Table 3:** Incidence of PVR and procedural parameters in the case of initial ablation with and without strict adherence to the CLOSE protocol. Categorical variables are reported as frequency and percentage. Continuous variables are reported as median and inter-quartile range. Bold values indicate significance at *p* < 0.05. FPI = first-pass isolation, OR = odds ratio, PV = pulmonary vein, PVI = pulmonary vein isolation, PVR = pulmonary vein reconnection, RF = radiofrequency.

		CLOSE (*n* = 38)	No CLOSE (*n* = 62)	*p*-Value	OR
Number of PVRs		39/149 (26.1%)	153/224 (68.3%)	**<0.0001**	0.16
Patients with all PVs isolated		15/38 (39.5%)	2/62 (3.2%)	**<0.0001**	19.57
Procedure time (min)		81 (70–100.8)	85 (70–105)	0.8258	
Left atrial dwell time (min)		60 (53.25–73.25)	58 (41–67)	0.0844	
Fluoroscopy time (min)		4.7 (2.5–8.6)	5.8 (3.9–13)	**0.0467**	
Dose area product (uGym^2^)		152.6 (95–320)	232 (160–460)	**0.0334**	
Catheter, *n* (%)	ST QDOT	31 (82) 7 (18)	51 (85) 9 (15)	0.7804	
Power, W		30 (30–45)	30 (30–40)	0.2368	
Number of RF applications, *n*		72 (64–80)	82 (67–116)	**0.0202**	
FPI, *n* (%)		28 (73.7)	8 (25)	**<0.0001**	
Baseline generator impedance, Ω		131.7 (125.8–142)	136.3 (127.9–1145)	0.2454	

**Table 4:** Univariate analyses for predictors of ≥1 PVR per patient. Bold values indicate significance (*p* < 0.05), * indicates relevant univariate variables (*p* < 0.1). AF = atrial fibrillation, BMI = body mass index, CI = confidence interval, FPI = first-pass isolation, HPSD = high-power short-duration, LAD = left atrial diameter, LVEF = left ventricular ejection fraction, OR = odds ratio, PV = pulmonary vein, PVI = pulmonary vein isolation, PVR = pulmonary vein reconnection.

	Univariate Logistic Regression Analysis for Predictors of at Least One PVR vs. All PVs Isolated per Patient		
	OR	95% CI	*p*-Value
Adherence to CLOSE protocol	0.0548	0.0082–0.2141	**0.0002** *
Catheter (QDOT)	0.3478	0.1029–1.274	0.0941 *
Power (HPSD)	0.4000	0.1296–1.225	0.0988 *
Bilateral FPI	0.0933	0.0138–0.3765	**0.0032** *
Baseline generator impedance ≥ 130 Ω	7.386	2.293–26.96	**0.0012** *
Mean PV perimeter, cm	1.184	0.8193–1.747	0.3635
Time to recurrence, month	0.9778	0.9443–1.013	0.2027
Time to repeat, month	0.9820	0.9520–1.014	0.2555
Age, year	0.9814	0.9339–1.026	0.4286
Sex (female)	1.007	0.3440–3.189	0.9907
Persistent AF	0.8949	0.3108–2.613	0.8359
BMI, kg/m^2^	1.049	0.9449–1.170	0.3756
LVEF, %	1.059	0.9857–1.141	0.1169
LAD, mm	0.9844	0.8990–1.074	0.7242

**Table 5:** Multivariable analyses for predictors of ≥1 PVR per patient. Bold values indicate significance (*p* < 0.05). CI = confidence interval, FPI = first-pass isolation, HPSD = high-power short-duration, OR = odds ratio, PV = pulmonary vein, PVR = pulmonary vein reconnection.

	Multivariable Logistic Regression for Predictors of at Least One PVR vs. All PVs Isolated per Patient		
	OR	95% CI	*p*-Value
Adherence to CLOSE protocol	0.0546	0.005–0.614	**0.0187**
Catheter (QDOT)	0.2335	0.177–103.110	0.3705
Power (HPSD)	0.4477	0.054–3.744	0.4576
Bilateral FPI	0.1159	0.010–1.354	0.0862
Baseline generator impedance ≥ 130 Ω	16.09	1.688–153.684	**0.0157**

## Data Availability

The data underlying this article cannot be publicly shared due to privacy and ethical considerations as per General Data Protection Regulation. Upon reasonable request, the corresponding author will provide access to the data.
